# Evidence of heat sensitivity in people with Parkinson’s disease

**DOI:** 10.1007/s00484-024-02658-w

**Published:** 2024-04-11

**Authors:** Andrew P. Hunt, Aline Souza Pagnussat, Alexander Lehn, Daniel Moore, Daniel Schweitzer, E-Liisa Laakso, Ewald Hennig, Meg E. Morris, Graham Kerr, Ian Stewart

**Affiliations:** 1 School of Biomedical Sciences, Faculty of Health, Queensland University of Technology (QUT), Brisbane, 4059 Australia; 2 Department of Physical Therapy, Universidade Federal de Ciências da Saúde de Porto Alegre, Porto Alegre, Brazil; 3 Department of Physical Therapy, Georgia State University, Atlanta, USA; 4 Princess Alexandra Hospital, Brisbane, QLD Australia; 5 School of Medicine, The University of Queensland, Brisbane, QLD Australia; 6 School of Exercise and Nutrition Sciences, Faculty of Health, Deakin University, Melbourne, Australia; 7 Centre for Neurosciences, Mater Hospital, Brisbane, QLD Australia; 8 Wesley Hospital, Brisbane, QLD Australia; 9 Mater Research Institute, The University of Queensland, Brisbane, QLD Australia; 10 Menzies Health Institute Queensland, Griffith University, Gold Coast, QLD Australia; 11 School of Exercise and Nutrition Sciences, Faculty of Health, Queensland University of Technology (QUT), Brisbane, 4059 Australia; 12 The Victorian Rehabilitation Centre and ARCH, La Trobe University, Bundoora, 3086 Australia

**Keywords:** Parkinson’s disease, Heat intolerance, Quality of life, Hot temperature, Activities of daily living, Hyperhidrosis, Climate change

## Abstract

Examining how heat affects people with Parkinson’s disease is essential for informing clinical decision-making, safety, well-being, and healthcare planning. While there is evidence that the neuropathology associated with Parkinson’s disease affects thermoregulatory mechanisms, little attention has been given to the association of heat sensitivity to worsening symptoms and restricted daily activities in people with this progressive disease. Using a cross-sectional study design, we examined the experiences of people diagnosed with Parkinson’s disease in the heat. Two-hundred and forty-seven people completed an online survey (age: 66.0 ± 9.2 years; sex: male = 102 (41.3%), female = 145 (58.7%)), of which 195 (78.9%) reported becoming more sensitive to heat with Parkinson’s disease. Motor and nonmotor symptoms worsened with heat in 182 (73.7%) and 203 (82.2%) respondents, respectively. The most commonly reported symptoms to worsen included walking difficulties, balance impairment, stiffness, tremor, fatigue, sleep disturbances, excess sweating, difficulty concentrating, and light-headedness when standing. Concerningly, over half indicated an inability to work effectively in the heat, and nearly half reported that heat impacted their ability to perform household tasks and social activities. Overall, heat sensitivity was common in people with Parkinson’s disease and had a significant impact on symptomology, day-to-day activities and quality of life.

**Supplementary Information:**

The online version contains supplementary material available at 10.1007/s00484-024-02658-w.

## Introduction

Globally, over six million people live with Parkinson’s disease (GBD 2016 Neurology Collaborators 2019; Dorsey et al. 2018a), and the incidence is climbing (Dorsey et al. 2018b). Disability-adjusted life years (DALYs), estimated to be over three million, and deaths resulting from Parkinson’s disease have more than doubled from 1990 to 2016 (Dorsey et al. 2018a, b; GBD 2016 Neurology Collaborators 2019). Although primarily recognised as a movement disorder evidenced by bradykinesia, tremor, rigidity, postural instability, and gait disturbances, a range of nonmotor symptoms, including depression, anxiety, apathy, cognitive impairment, loss of sense of smell, and sleep disturbance are also common (Politis et al. 2010; Asahina et al. 2013; Verbaan et al. 2007; Bloem et al. 2021). Thermoregulatory dysfunction stemming from central and peripheral neural degeneration has also been observed (Coon and Low 2018).

Within the brain, Lewy bodies in the hypothalamus and alpha-synuclein containing Lewy bodies in the medulla are thought to affect the sympathetic nervous system’s role in vasomotor and sudomotor tone, the primary avenues for temperature regulation (Coon and Low 2018; Bongioanni et al. 2021). Additionally, small- and large-fibre peripheral neuropathies reduce autonomic innervation of the blood vessels, sweat glands, and erector pili muscles (Dabby et al. 2006; Donadio et al. 2014), thus altering the effectiveness of skin blood flow and sweating responses. Changes in sensory thresholds also impact temperature perception and thermal information processing (Conte et al. 2013). While there is evidence that neurodegeneration due to Parkinson’s disease affects thermoregulatory mechanisms, little attention has been given to a sensitivity to heat experienced by people living with Parkinson’s disease.

Heat sensitivity, commonly described as a worsening of symptoms and restricted daily activities when exposed to heat, has been extensively reported among people with other neurological diseases, including multiple sclerosis (Christogianni et al. 2018, 2022) and Alzheimer’s disease (Amiri et al. 2021). People with multiple sclerosis report that heat sensitivity contributes to heightened levels of fatigue, weakness, poorer walking ability and balance difficulties, and an impaired ability to walk, concentrate, think and perform physical activity (Christogianni et al. 2022). In Alzheimer’s disease, cognitive symptoms, including agitation, hallucinations, irritability, sleep disturbances, anxiety and depression reportedly worsen in higher ambient temperatures (Amiri et al. 2021). Although heat intolerance is broadly acknowledged for people with Parkinson’s disease (Linares et al. 2016), specific effects on motor and nonmotor symptoms have not been characterised or investigated in research literature (Amiri et al. 2021).

The need to better understand how heat sensitivity affects Parkinson’s disease is underscored by the effects of climate change on health, particularly for vulnerable populations (Romanello et al. 2022). The greater frequency and intensity of heat waves due to climate change is concerning as increased morbidity and mortality for people living with Parkinson’s disease during heat waves has been documented (Linares et al. 2016; Lin et al. 2023). Incorporating heat sensitivity as part of clinical assessments and health care action plans may enable clinicians and individuals living with Parkinson’s disease to prepare for and manage the increasing risks posed by hot weather and aligns with achieving the sustainable development goal of the United Nations for health and well-being (United Nations 2015). Therefore, this study aimed to elucidate the experience of heat sensitivity among people diagnosed with idiopathic Parkinson’s disease.

## Methods

A cross-sectional study design and survey were used with people diagnosed with Parkinson’s disease to understand their experiences with heat. An online survey was distributed internationally via social media and Parkinson’s disease organisations and community groups between November 2020 and April 2023. Prospective participants could read information about the survey before commencing, and completion and submission of the survey constituted consent to participate (all responses were anonymous). The study was approved by the Queensland University of Technology Human Research Ethics Committee (approval 3476), and it has been reported in accordance with STROBE guidelines for cross-sectional studies (von Elm et al. 2007).

We developed a questionnaire to explore the issues relating to heat sensitivity for people with Parkinson’s disease (Supplementary materials). The scientific literature and online Parkinson’s disease community forums were explored for information related to heat sensitivity and intolerance. Other relevant questionnaires for heat sensitivity in multiple sclerosis (Flensner et al. 2011; Summers et al. 2012) and the general population were also consulted (Van Someren et al. 2016). Once drafted, the survey was further developed in consultation with a panel of experts, including two neurologists, a physiotherapist, and two researchers, all of whom specialise in working with people with Parkinson’s disease. The feedback received resulted in a range of amendments that improved question clarity and response options.

The questionnaire comprised five main sections. Section 1 collected demographic information (age, sex, height, weight, and location). In Sect. 2, respondents confirmed they had been diagnosed with Parkinson’s disease by a medical professional (e.g., a movement disorders specialist, neurologist, or geriatrician). Further medical information such as age at diagnosis, prescribed medications for Parkinson’s disease, treatment by deep brain stimulation, other medical conditions and prescribed medications was also collected. This section also asked respondents to answer the question (Yes/No) “Have you become more sensitive to the heat with Parkinson’s disease?”

Health status related to Parkinson’s disease was assessed in Sect. 3 with the Parkinson’s disease questionnaire 8-item scale (PDQ-8) (Jenkinson et al. 1997). This questionnaire asks respondents to rate 8 items on a 5-point Likert scale (never, occasionally, sometimes, often, always). The responses are summed to calculate the total score from 0 to 32 (PDQ-8) and can be standardised to a scale from 0 to 100 (PDQ-8SI).

In Sect. 4, heat sensitivity was reported using ten statements (Yes/No) following the question, “What happens to you when you get too hot?” These statements were adapted from a similar study among people with multiple sclerosis (Summers et al. 2012):

1. Nothing, I cope just fine (Nothing);
2. I lack energy and require more rest (Lack Energy);
3. Apart from fatigue, my other symptoms of Parkinson’s disease become worse (Worse PD Symptoms);
4. I am unable to participate in my usual social activities (time with family or friends (Unable – Social Activities);
5. I am unable to do my usual household duties (e.g., cleaning, cooking, etc. (Unable - Household Tasks);
6. I am unable to work effectively (Unable - Work Effectively);
7. I am unable to look after myself in the usual manner (Unable - Self Care);
8. I need more medication to cope (More Medication);
9. I have felt sufficiently unwell to require a doctor or other health professional (Visit Doctor);
10. I have been hospitalised because of heat (Hospitalised).

Access to air conditioning was investigated in Sect. 5. Respondents were asked if they used air conditioners at home to keep cool on hot days or nights. Those answering yes were asked at what outdoor temperature they turn their air conditioning on, and whether they receive a subsidy or rebate for the cost of cooling their home.

In the remainder of the questionnaire, respondents rated a range of statements on a 5-point Likert scale (never, rarely, sometimes, often, always) relating to their experiences in heat. The following five of those statements are reported in this paper:

1. I sweat too much in the heat;
2. My movement symptoms of Parkinson’s disease get worse in the heat;
3. My non-movement symptoms of Parkinson’s disease get worse in the heat, e.g., fatigue, depression;
4. My Parkinson’s medication is less effective in the heat;
5. My OFF periods (times when medication is less effective) are made worse by the heat.

Those indicating a response ‘greater than never’ were prompted to indicate in which body regions they experience excessive sweating and which symptoms (from a given list) become worse in the heat. Finally, an optional question asked respondents to write in their own words how heat affects them. However, these responses will be reported elsewhere.

### Data analysis

Continuous variables were assessed for a normal distribution via a Shapiro Wilk test and summarised as means (± SD) when normally distributed or median [interquartile range] when not normally distributed. Categorical variables were summarised as counts and percentages (%). Normally distributed continuous variables were compared with t-tests with equal variance assumed (students t-test). Non-normally distributed continuous variables were compared with the Wilcoxon rank-sum test (with continuity correction). Pearson’s chi-squared tests with Yate’s continuity correction were used for the comparison of categorical variables. Where required, post hoc testing of significant chi-squared outcomes was performed by comparing standardized residuals with a critical value determined by Bonferroni correction to the alpha level. Statistical significance was accepted at *p* < 0.05. Data analysis and visualisation were performed using R Studio (version 1.3.1093) with the following packages: tidyr, dplyr, rlang, epiDisplay, userfrindelyscience, ggplot2, gridExtra, stringr, and psych.

## Results

### Participants

Two-hundred and forty-seven people diagnosed with Parkinson’s disease completed the survey (age: 66.0 ± 9.2 years; sex: male = 102 (41.3%), female = 145 (58.7%); height: 168 [161–177] cm; body mass: 76 [64–89] kg; BMI: 25.9 [22.7–31.1] kg/m^2^). Respondents were from Australia (*n* = 117, 47.4%), the United Kingdom (*n* = 84, 34.0%), Canada (*n* = 24, 9.7%), the United States of America (*n* = 19, 7.7%), and other countries (New Zealand = 1, Brazil = 1, France = 1).

The mean age at diagnosis was 59.3 ± 10.1 years, and respondents had lived with Parkinson’s disease for a median of 5 [3–9] years. Health status related to Parkinson’s disease, as indicated by the PDQ-8 and PDQ-8SI, was 9 [6–13] and 28.1 [18.8–40.6], respectively. The vast majority (96%) of respondents were taking prescription medication for treating their Parkinson’s disease (Supplementary Table 1), and 17 (6.9%) of respondents were currently being treated by deep brain stimulation. A range of comorbidities was also reported by respondents (Supplementary Table 2).

### Heat sensitivity

One hundred and ninety-five (78.9%) respondents self-reported becoming more sensitive to heat with Parkinson’s disease. Those indicating becoming heat-sensitive were significantly younger, were younger at the time of their diagnosis, had higher body mass index values, and had greater severity of Parkinson’s disease symptoms (PDQ-8 and PDQ-8SI) compared to those who weren’t heat sensitive (Table 1). The proportion of heat-sensitive respondents did not vary by sex but did vary with location (Table 1), being reported significantly more frequently in Australia compared to the United Kingdom (countries with low sample sizes were excluded).

**Table 1:** Comparisons of respondent characteristics by heat sensitivity groups

	Heat Sensitive - No (*n* = 52)	Heat Sensitive - Yes (*n* = 195)	Test statistic	p-value
Age (years)	69.2 ± 10.1	65.2 ± 8.9	t = 2.81	**0.0054**
Sex *				
Male	27 (26%)	75 (74%)	χ^2^ = 2.5385	0.1111
Female	25 (17%)	120 (83%)		
Height (cm)	168 [164–178]	168 [160–177]	W = 5356	0.5325
Body Mass (kg)	73 [63–86]	77 [65–90]	W = 4836	0.135
BMI (kg/m^2^)	23.7 [21.9–28.8]	26.4 [23.1–31.5]	W = 4073	**0.0294**
Country *				
Australia	14 (10%)	103 (90%)	χ^2^ = 15.84	**< 0.001** ^**+**^
United Kingdom	29 (35%)	55 (65%)		
North America	7 (16%)	36 (84%)		
Age at diagnosis (years)	62.5 ± 10.0	58.5 ± 10.0	t = 2.570	**0.0108**
Years with PD (years)	4.5 [3.0–8.0]	5.0 [3.0–9.0]	W = 4838	0.6118
PDQ-8	7.0 [5.0–10.0]	10.0 [6.0–14.0]	W = 3652	**0.0019**
PDQ-8SI	21.9 [15.6–31.3]	31.3 [18.8–43.8]	W = 3652	**0.0019**
Deep brain stimulation *				
Yes	2 (12%)	15 (88%)	χ^2^ = 0.95	0.33
No	50 (22%)	180 (78%)		

* Row percentages; ^**+**^ post hoc analysis showed Australia was significantly different from the United Kingdom; BMI, body mass index; PD, Parkinson’s disease; PDQ-8, Parkinson’s disease Questionnaire-8 item; PDQ-8SI, Parkinson’s disease Questionnaire-8 item standardised

**Fig. 1:**
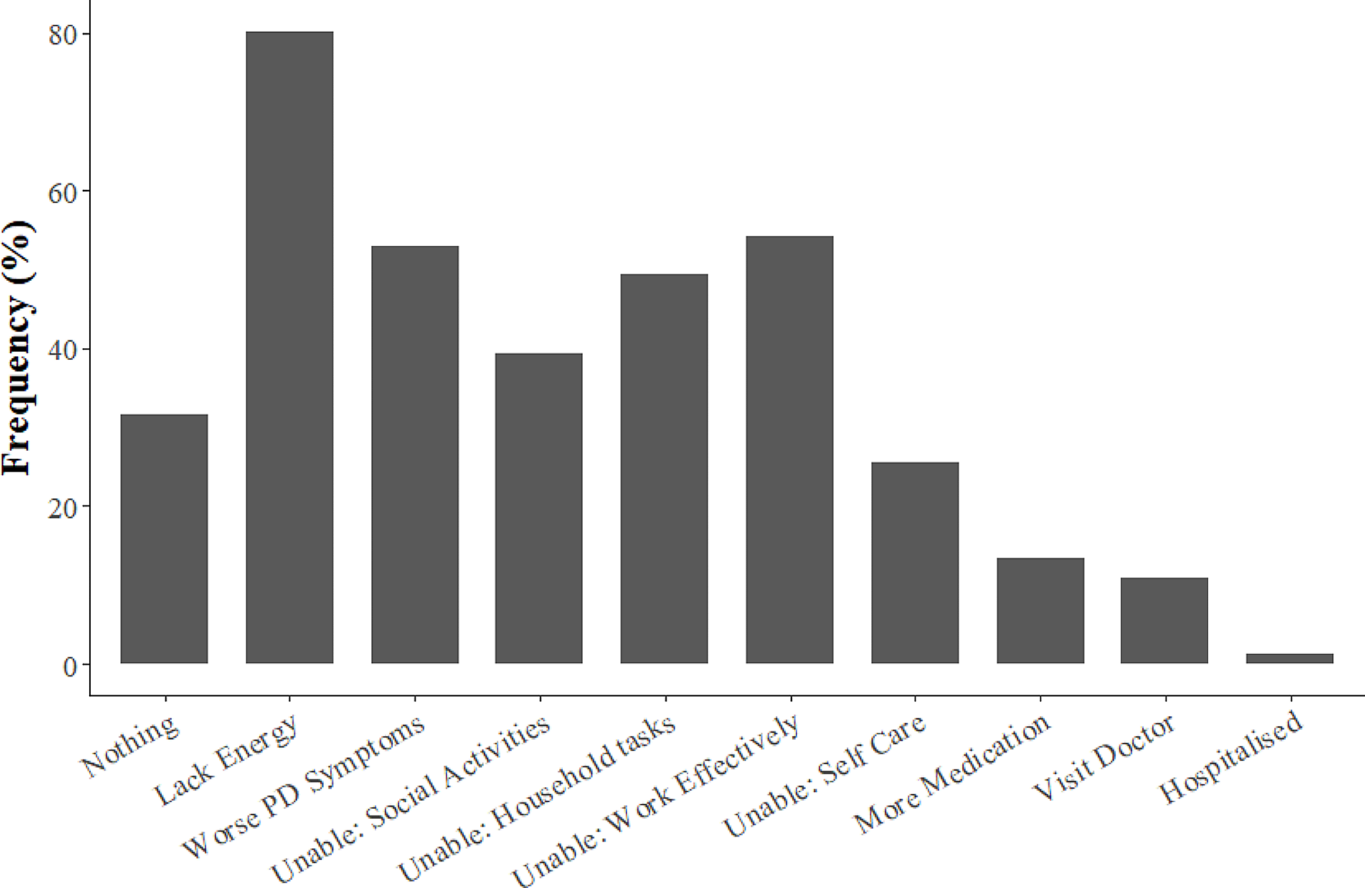
Indicators of heat sensitivity

80% of all respondents reported experiencing a lack of energy when they get too hot (Fig. 1). More than 50% also experienced a worsening of their Parkinson’s disease symptoms and an inability to work effectively. The inability to engage in usual social activities and perform household duties was reported by ~ 40% and ~ 50% of respondents, respectively. Comparing between groups (Table 2), people with heat sensitivity were significantly more likely to lack energy and experience worse Parkinson’s disease symptoms when they get too hot. Furthermore, heat sensitivity was associated with the inability to participate in social activities, perform household tasks, work effectively, and look after oneself in the usual manner (Table 2). In contrast, heat sensitive people were significantly less likely to agree with the statement “Nothing, I cope just fine.”

Compared to males, a significantly higher proportion of females reported a lack of energy in the heat (74% vs. 85%, χ^2^ = 4.12, *p* = 0.042) and an inability to perform household tasks (39% vs. 57%, χ^2^ = 6.52, *p* = 0.011). The other heat sensitivity indicators did not differ between the sexes.

**Table 2:** Indicators of heat sensitivity compared between self-reported heat sensitivity groups

Item	Response	Heat Sensitive - No	Heat Sensitive - Yes	Chi-squared (χ^2^)	P-value
Nothing, I cope just fine					
	No	16 (31%)	153 (78%)	41.04	**< 0.001**
	Yes	36 (69%)	42 (22%)		
Lack Energy					
	No	25(48%)	24 (12%)	30.82	**< 0.001**
	Yes	27 (52%)	171 (88%)		
Worse PD Symptoms					
	No	39 (75%)	77 (39%)	19.39	**< 0.001**
	Yes	13 (25%)	118 (61%)		
Unable: Social Activities					
	No	44 (85%)	106 (54%)	14.52	**< 0.001**
	Yes	8 (15%)	89 (46%)		
Unable: Household tasks					
	No	39 (75%)	86 (44%)	14.48	**< 0.001**
	Yes	13 (25%)	109 (56%)		
Unable: Work effectively					
	No	38 (73%)	75 (38%)	18.45	**< 0.001**
	Yes	14 (27%)	120 (62%)		
Unable: Self-care					
	No	46 (88%)	138 (71%)	5.86	**0.015**
	Yes	6 (12%)	57 (29%)		
More Medication					
	No	48 (92%)	166 (85%)	1.26	0.262
	Yes	4 (8%)	29 (15%)		
Visit Doctor					
	No	50 (96%)	170 (87%)	2.54	0.111
	Yes	2 (4%)	25 (13%)		

Data represent counts and column percentages. Statistically significant p-values (< 0.05) are presented in bold font

Motor and nonmotor symptoms of Parkinson’s disease were reported to worsen in heat (rated ‘greater than never’) by 182 (73.7%) and 203 (82.2%) respondents, respectively (Supplementary Fig. 1). The most common (> 30% of respondents) motor symptoms to worsen in the heat included walking difficulties, balance impairments, stiffness, and tremor (Table 3). Fatigue, sleeping difficulties, excess sweating, difficulty concentrating, and light-headedness when standing were the most commonly reported nonmotor symptoms (> 30% of respondents) (Table 3). Excessive sweating in the heat (rated ‘greater than never’) was reported by 206 (83.4%) respondents. Excessive sweating was experienced in various body regions, most commonly on the face, head, and neck (Table 4).

**Table 3:** Frequency of worsening motor and nonmotor symptoms in heat

Motor Symptom	Frequency	Nonmotor Symptom	Frequency
Walking difficulties	94 (38.1%)	Fatigue	174 (70.4%)
Balance impairments	93 (37.7%)	Sleeping disturbances	123 (49.8%)
Stiffness	92 (37.2%)	Excessive sweating	104 (42.1%)
Tremor	83 (33.6%)	Difficulty concentrating	100 (40.5%)
Difficulty dressing	48 (19.4%)	Lightheaded when standing	92 (37.2%)
Freezing while walking	35 (14.2%)	Anxiety	62 (25.1%)
Chewing/swallowing difficulties	21 (8.5%)	Memory problems	62 (25.1%)
		Frequent urination	58 (23.5%)
		Urgent urination	55 (22.3%)
		Constipation	46 (18.6%)
		Dribbling	44 (17.8%)
		Depression	33 (13.4%)
		Taste/smell problems	26 (10.5%)

Data represent counts and percentages of the total sample

**Table 4:** Frequency of excessive sweating on various body regions

Body region	Frequency
Whole body	58 (23.5%)
Face	101 (40.9%)
Head	84 (34.0%)
Neck	81 (32.8%)
Upper back	68 (27.5%)
Lower back	57 (23.1%)
Chest	57 (23.1%)
Feet	35 (14.2%)
Upper arms	33 (13.4%)
Thighs	32 (13.0%)
Abdomen	31 (12.6%)
Hands	24 (9.7%)
Pelvis	24 (9.7%)
Lower legs	11 (4.5%)
Lower arms	9 (3.6%)

Data represent counts and percentages of the total sample

### Air conditioning

One hundred and forty-one (57%) respondents used air conditioning to cool their home, differing by country (Australia: 101 (86%); United Kingdom: 6 (7%); North America: 33 (77%); χ2 = 133.36, *p* < 0.001). Those using air conditioners reported turning their air conditioner on when outdoor air temperatures were 20–24 °C (*n* = 19, 7.7%)), 25–29 °C (*n* = 64, 25.9%)), 30–34 °C (*n* = 65, 26.3%)), or 35–39 °C (*n* = 21, 8.5%)). Only 26 out of 96 Australian respondents with air-conditioning indicated receiving a government rebate or subsidy for the cost of cooling their home, from Queensland (7 out of 26), Victoria (8 out of 24), New South Wales (4 out of 16), South Australia (2 out of 2), Western Australia (6 out of 25), Tasmania (0 out of 3) and Australian Capital Territory (0 out of 2). No respondents in other countries received a rebate for cooling their homes.

### Medications and comorbidities

Respondents indicated taking a range of prescribed medications for treating Parkinson’s disease (Supplementary Table 1). There were no statistically significant associations between prescription medications and self-reported heat sensitivity (Supplementary Table 1). One hundred and fifty-six (63%) respondents reported their medication was less effective in the heat and 155 (63%) indicated that their off periods become worse in the heat (Supplementary Fig. 1).

The respondents reported a range of comorbidities (Supplementary Table 2). Hypertension, depression, anxiety, and arthritis were each reported by approximately 25% of respondents. The proportion of people with heat sensitivity with arthritis or anxiety was significantly higher than among people without self-reported heat sensitivity (Supplementary Table 2). One hundred and thirty-four (54%) respondents were taking prescription medication for their comorbidities.

## Discussion

Our exploratory survey reveals that many people with Parkinson’s disease experience heat sensitivity. As well as increasing fatigue, heat exacerbates both motor and nonmotor symptoms and impacts the ability to perform activities of daily living. It is recommended that clinicians consider heat sensitivity when assessing and treating people with Parkinson’s disease, and government policies and international recommendations (such as those of the World Health Organisation) may require revision to support and safeguard these vulnerable community members during hot weather.

Rising average temperatures are exposing humans to hotter weather around the world (Romanello et al. 2022). Heatwaves, prolonged periods of excessive heat (Perkins and Alexander 2013), are increasing in frequency, intensity, and duration (Perkins-Kirkpatrick and Lewis 2020). Excessive heat varies by location. For example, heatwaves in the UK occur at temperatures above 26–28 °C (depending on county thresholds 1), but in Australia, heatwaves are defined by an excess heat factor 2, with temperatures generally in the high 30s and potentially high humidity. Hot weather and heatwaves have a significant impact on human health, particularly for vulnerable populations (Romanello et al. 2022). As such, a greater understanding of how heat affects symptoms and day-to-day life for people with Parkinson’s disease may enable clinicians and individuals to prepare for and manage these events.

Thermoregulatory dysfunction in Parkinson’s disease results from both central and peripheral neuropathology resulting in sweating and skin blood flow alterations (Dabby et al. 2006; Donadio et al. 2014). Medications prescribed for treating Parkinson’s disease can also adversely impact thermoregulation (Wee et al. 2023). Impaired body temperature regulation reduces tolerance to hot weather, evidenced by increased morbidity and mortality for people with Parkinson’s disease in heat waves (Linares et al. 2016; Lin et al. 2023), but little attention has been devoted to understanding how heat exposure affects symptomology and activities of daily living. Some research has focused on seasonal variations in symptomology and found minimal changes in the summer months (Postuma et al. 2005). However, only examining seasonal variation does not account for unseasonably hot weather or heat waves that cause acute heat exposures. Additionally, seasonal changes in behaviours and potential adjustments in medication regimens may have masked potential changes in symptomology. For example, a relationship between levodopa prescriptions and seasonal temperature has been observed, suggesting changes in symptomology might increase medication requirements in summer (Rowell et al. 2017). Considering these gaps in the literature, our survey provides additional evidence of heat sensitivity among many people with Parkinson’s disease, shown by worsening motor and nonmotor symptoms in heat (Table 3). Heat sensitivity also negatively impacted activities of daily living, such as the ability to work effectively, perform household tasks, attend social activities, and for some respondents (~ 25%) an inability to look after themselves in their usual manner.

Several insights regarding heat sensitivity can be gleaned from the results of our survey. Heat sensitivity was associated with higher PDQ8 scores, an assessment of health status that also relates to the Hoehn and Yahr stages of Parkinson’s disease severity (Jenkinson et al. 1997). Similar to other autonomic impairments (Merola et al. 2018), heat sensitivity was associated with overall disease progression and severity. In addition, heat sensitivity was more common in respondents from Australia than the United Kingdom (Table 1). Heat sensitivity in multiple sclerosis is also more common among people in Australia than in Sweden (Summers et al. 2012; Flensner et al. 2011). These findings suggest heat sensitivity may be more prevalent in persons habitually exposed to warmer climates. With the rising incidence of neurological disorders (Dorsey et al. 2018b; GBD 2016 Neurology Collaborators 2019), a trend that may be associated with climate change (Bongioanni et al. 2021), these findings regarding geographical location and climate are concerning and require further exploration. Finally, much of the previous research on temperature regulation in people with Parkinson’s disease has focused on sweating. Similar to previous research (Swinn et al. 2003; Coon and Low 2018), our findings show that excessive sweating is common in this cohort (Table 4). Excessive sweating can also have detrimental effects on physical, social, and emotional well-being (Swinn et al. 2003).

Our findings are relevant for clinical practice, raising awareness of the issue of heat sensitivity and related medications, comorbidities, and government support schemes. Education about the effects of heat on both motor and nonmotor symptoms, as well as the establishment of heat avoidance or cooling strategies, are important ways clinicians can support heat sensitive people with Parkinson’s disease. For example, balance and gait impairments and light-headedness are predictive of falls (Kerr et al. 2010; Cole et al. 2010). Therefore, the risk of falls may be elevated in heat sensitive individuals during hot weather. Cooling and heat avoidance strategies might consider being active in cooler times of day, wearing loose-fitting clothing, fanning or air-conditioning, or cool water baths (Christogianni et al. 2022). Additionally, adjusting therapy settings and medication regimens, closely monitoring patients during hot weather, and developing emergency plans for heatwaves are measures that may significantly contribute to improving overall care and well-being.

While no prescribed medications for treating the symptoms of Parkinson’s disease were associated with heat sensitivity (Supplementary Table 1), it was common for respondents to indicate that medication became less effective in heat (Supplementary Fig. 1). This finding supports previous research showing an increase in levodopa prescriptions during summer (Rowell et al. 2017). Aside from potential effects on heat sensitivity, dopamine replacement agents and anticholinergics commonly prescribed for Parkinson’s disease adversely affect thermoregulation (Wee et al. 2023). Taken together, these findings suggest medication requirements may be an important aspect to consider during heat health action planning for patients, clinicians, and caregivers. Further research should investigate the effectiveness of Parkinson’s disease medications during periods of heat exposure, and their impact on heat sensitivity and thermoregulation.

In our investigation, anxiety and arthritis were more common in those with heat sensitivity (Supplementary Table 2). Anxiety is common in Parkinson’s disease (Broen et al. 2016) and it is more frequent among those who experience excessive sweating (van Wamelen et al. 2019). Our finding that heat sensitivity also influences anxiety is concerning given its negative impact on quality of life (Chuquilín-Arista et al. 2020). Regarding arthritis, previous research has demonstrated an influence of hot weather on arthritis pain (Aikman 1997; Timmermans et al. 2015), and access to physical therapy for arthritis pain increases in heat (Wu et al. 2022).

In the present study, just over half of the respondents used air-conditioning in their homes. In Australia, some respondents received a government subsidy for the cost of cooling their homes. Such policies should be made widely accessible to support people with neurological impairments that increase sensitivity and intolerance to heat (Ito et al. 2018; Summers et al. 2012). While air conditioning effectively reduces heat strain by controlling the environmental conditions, it is expensive for individuals and increasing widespread dependence due to climate change has negative impacts on the broader community (Ito et al. 2018; Farbotko and Waitt 2011; Jay et al. 2021). As such, the effectiveness of other cooling strategies should also be investigated.

Several limitations should be noted. A range of ‘Parkinsonian plus’ conditions, including progressive supranuclear palsy, multiple systems atrophy and lewy body disease, may share the same overlapping clinical features but differ in the underlying neuropathology from idiopathic Parkinson’s disease. The present study did not account for these potential differences. Siderowf et al. (Siderowf et al. 2023) showed that new techniques have the potential to differentiate people with Parkinson’s disease from healthy controls. Future clinical trials will warrant the inclusion of these diagnostic tests to confirm the diagnosis of PD, as advised by the Parkinson’s Progression Marker Initiative (Siderowf et al. 2023).

Another limitation was that various types of heat exposure were not differentiated in participant responses. Heat exposure may be from external (environmental) or internal (metabolic) heat, which may have different physiological effects regarding heat sensitivity. The impact of environmental heat may depend on whether it is habitual or an acute weather event and may also vary with humidity, sunlight exposure, and acclimatisation status. Further research should elucidate the heat exposure triggers for heat sensitivity. Finally, further research should also evaluate cold sensitivity in this population.

## Conclusions

Heat sensitivity affects many people living with Parkinson’s disease, exacerbating symptoms and impairing their ability to perform activities of daily living. Patients, clinicians, and caregivers are recommended to consider the effects of heat when developing health care action plans. Given rapid global climate change, heat policy reform is required at local, state, and national government levels, as well as internationally, to provide support schemes accessible for these vulnerable members of the community.

### Electronic supplementary material

Below is the link to the electronic supplementary material.

Supplementary Material 1

Supplementary Material 2

Supplementary Material 3

Supplementary Material 4

## Data Availability

The data that support the findings of this study are available from the corresponding author upon reasonable request.

## Footnotes

1 The Met Office, UK: https://www.metoffice.gov.uk/weather/learn-about/weather/types-of-weather/temperature/heatwave. Accessed 26th February 2024.

2 The Bureau of Meteorology, Australia: http://www.bom.gov.au/australia/heatwave/knowledge-centre/understanding.shtml. Accessed 26th February 2024.
