# Supplementary material for: Evidence of heat sensitivity in people with Parkinson’s disease
Source: Int J Biometeorol. 2024 Apr 11;68(6):1169–78. doi: 10.1007/s00484-024-02658-w (PMC11108869; doi:10.1007/s00484-024-02658-w)
Supplement: Supplementary file 1 — Supplementary Material 1 [file 484_2024_2658_MOESM1_ESM.docx]

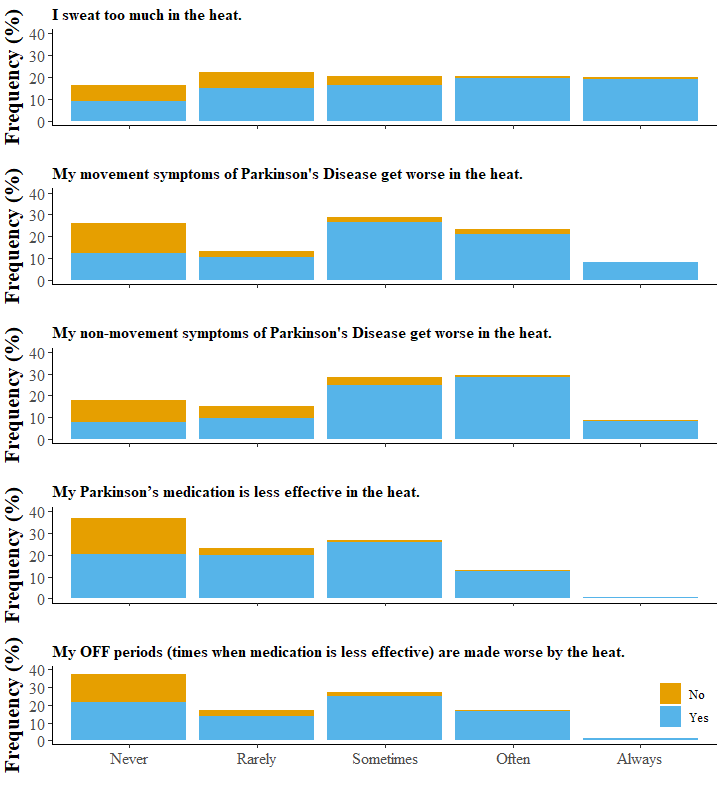


**Supplementary Figure 1: Rating frequencies for five statements about sweating, Parkinson’s disease symptoms, and medications, grouped by heat sensitivity status (yes/no)**
