# Supplementary material for: Evidence of heat sensitivity in people with Parkinson’s disease
Source: Int J Biometeorol. 2024 Apr 11;68(6):1169–78. doi: 10.1007/s00484-024-02658-w (PMC11108869; doi:10.1007/s00484-024-02658-w)
Supplement: Supplementary file 2 — Supplementary Material 2 [file 484_2024_2658_MOESM2_ESM.docx]

**Supplementary Table 1: Frequency of prescription medications taken by respondents compared between self-reported heat sensitivity groups.**

| **Medications** | **Response** | **Heat Sensitive - No** | **Heat Sensitive - Yes** | **Chi-squared (χ^2^)** | **P-value** |
| --- | --- | --- | --- | --- | --- |
| Madopar (Levodopa-Benserazide) | |  |  |  |  |
|  | No | 32 (62%) | 114 (58%) | 0.059 | 0.809 |
|  | Yes | 20 (38%) | 81 (42%) |  |  |
| Sinemet (Carbidopa-Levodopa Oral) | |  |  |  |  |
|  | No | 37 (71%) | 129 (66%) | 0.266 | 0.606 |
|  | Yes | 15 (29%) | 66 (34%) |  |  |
| Azilect (Rasagiline) | |  |  |  |  |
|  | No | 40 (77%) | 156 (80%) | 0.087 | 0.769 |
|  | Yes | 12 (23%) | 39 (20%) |  |  |
| Sinemet CR (Carbidopa-Levodopa Oral, Extended Release) | | | |  |  |
|  | No | 47 (90%) | 158 (81%) | 1.928 | 0.165 |
|  | Yes | 5 (10%) | 37 (19%) |  |  |
| Stalevo (Carbidopa, Levodopa, and Entacapone) | | |  |  |  |
|  | No | 48 (92%) | 171 (88%) | 0.471 | 0.492 |
|  | Yes | 4 (8%) | 24 (12%) |  |  |
| Sifrol ER or Mirapex ER (Pramipexole) | | |  |  |  |
|  | No | 47 (90%) | 171 (88%) | 0.086 | 0.769 |
|  | Yes | 5 (10%) | 24 (12%) |  |  |
| Sifrol, Mirapex or Mirapexin (Pramipexole) | | |  |  |  |
|  | No | 50 (96%) | 175 (90%) | N/A | N/A |
|  | Yes | 2 (4%) | 20 (10%) |  |  |
| Neupro (Rotigotine) |  |  |  |  |  |
|  | No | 51 (98%) | 180 (92%) | N/A | N/A |
|  | Yes | 1 (2%) | 15 (8%) |  |  |
| Lodosyn (Carbidopa) |  |  |  |  |  |
|  | No | 49 (94%) | 195 (100%) | N/A | N/A |
|  | Yes | 3 (6%) | 0 (0%) |  |  |
| Duopa or Duodopa (Carbidopa-Levodopa Intestinal Gel) | | |  |  |  |
|  | No | 52 (100%) | 193 (99%) | N/A | N/A |
|  | Yes | 0 (0%) | 2 (1%) |  |  |
| No prescription medication |  |  |  |  |  |
|  | No | 4 (8%) | 6 (3%) | N/A | N/A |
|  | Yes | 48 (92% | 189 (97%) |  |  |
| Other |  |  |  |  |  |
|  | No | 29 (56%) | 132 (68%) | 2.073 | 0.150 |
|  | Yes | 23 (44%) | 63 (32%) |  |  |

Data represent counts and column percentages. N/A: chi-squared assumption violated – expected cell counts below 5.
