# Supplementary material for: Evidence of heat sensitivity in people with Parkinson’s disease
Source: Int J Biometeorol. 2024 Apr 11;68(6):1169–78. doi: 10.1007/s00484-024-02658-w (PMC11108869; doi:10.1007/s00484-024-02658-w)
Supplement: Supplementary file 3 — Supplementary Material 3 [file 484_2024_2658_MOESM3_ESM.docx]

**Supplementary Table 2: Frequency of comorbidities compared between self-reported heat sensitivity groups.**

| **Comorbidities** | **Response** | **Heat Sensitive - No** | **Heat Sensitive - Yes** | **Chi-squared (χ^2^)** | **P-value** |
| --- | --- | --- | --- | --- | --- |
| Hypertension | No | 43 (83%) | 141 (72%) | 1.816 | 0.178 |
|  | Yes | 9 (17%) | 54 (28%) |  |  |
| Depression | No | 43 (83%) | 144 (74%) | 1.299 | 0.254 |
|  | Yes | 9 (17%) | 51 (26%) |  |  |
| Arthritis | No | 46 (88%) | 142 (73%) | 4.697 | **0.0302** |
|  | Yes | 6 (12%) | 53 (27%) |  |  |
| Anxiety | No | 47 (90%) | 139 (71%) | 7.061 | **0.0079** |
|  | Yes | 5 (10%) | 56 (29%) |  |  |
| Cancer | No | 44 (85%) | 181 (93%) | N/A | N/A |
|  | Yes | 8 (15%) | 14 (7%) |  |  |
| Heat disease | No | 50 (96%) | 186 (95%) | N/A | N/A |
|  | Yes | 2 (4%) | 9 (5%) |  |  |
| Diabetes | No | 51 (98%) | 186 (95%) | N/A | N/A |
|  | Yes | 1 (2%) | 9 (5%) |  |  |
| Lung disease | No | 51 (98%) | 188 (96%) | N/A | N/A |
|  | Yes | 1 (2%) | 7 (4%) |  |  |
| Kidney disease | No | 48 (92%) | 195 (100%) | N/A | N/A |
|  | Yes | 4 (8%) | 0 (0%) |  |  |
| Liver disease | No | 52 (100%) | 193 (99%) | N/A | N/A |
|  | Yes | 0 (0%) | 2 (1%) |  |  |
| Other | No | 37 (71%) | 144 (74%) | 0.0456 | 0.831 |
|  | Yes | 15 (29%) | 51 (26%) |  |  |

Data represent counts and column percentages. N/A: chi-squared assumption violated – expected cell counts below 5. Statistically significant p-values (<0.05) are presented in bold font.
