# Supplementary material for: Evidence of heat sensitivity in people with Parkinson’s disease
Source: Int J Biometeorol. 2024 Apr 11;68(6):1169–78. doi: 10.1007/s00484-024-02658-w (PMC11108869; doi:10.1007/s00484-024-02658-w)
Supplement: Supplementary file 4 — Supplementary Material 4 [file 484_2024_2658_MOESM4_ESM.pdf]

Heat Sensitivity - Parkinson's Disease (HSPD)

(1/10) General information about you

What is your current age (years)?

- 18
- 19
- 20
- 21
- 22
- 23
- 24
- 25
- 30
- 31
- 32
- 33
- 34
- 35
- 36
- 37
- 38
- 39
- 40
- 41
- 42
- 43
- 44
- 45
- 46
- 47
- 48
- 49

○ 50
○ 51
○ 52
○ 53
○ 54
○ 55
○ 56
○ 57
○ 58
○ 59
○ 60
○ 61
○ 62
○ 63
○ 64
○ 65
○ 66
○ 67
○ 68
○ 69
○ 70
○ 71
○ 72
○ 73
○ 74
○ 75
○ 76
○ 77
○ 78

- ○ 79
- ○ 80
- ○ 81
- ○ 82
- ○ 83
- ○ 84
- ○ 85
- ○ 86
- ○ 87
- ○ 88
- ○ 89
- ○ 90
- ○ 91
- ○ 92
- ○ 93
- ○ 94
- ○ 95
- ○ 96
- ○ 97
- ○ 98
- ○ 99
- ○ 100
- ○ 101
- ○ 102
- ○ 103
- ○ 104
- ○ 105
- ○ 106
- ○ 107

- ○ 108
- ○ 109
- ○ 110
- ○ 111
- ○ 112
- ○ 113
- ○ 114
- ○ 115
- ○ 116
- ○ 117
- ○ 118
- ○ 119
- ○ 120+

What is your height (centimetres)?

- O 140
- O 141
- O 142
- O 143
- O 144
- O 145
- O 146
- O 147
- O 148
- O 149
- O 150
- O 151
- O 152
- O 153
- O 154
- O 155
- O 156
- O 157
- O 158
- O 159
- O 160
- O 161
- O 162
- O 163
- O 164
- O 165
- O 166
- O 167

- ○ 168
- ○ 169
- ○ 170
- ○ 171
- ○ 172
- ○ 173
- ○ 174
- ○ 175
- ○ 176
- ○ 177
- ○ 178
- ○ 179
- ○ 180
- ○ 181
- ○ 182
- ○ 183
- ○ 184
- ○ 185
- ○ 186
- ○ 187
- ○ 188
- ○ 189
- ○ 190
- ○ 191
- ○ 192
- ○ 193
- ○ 194
- ○ 195
- ○ 196

○	197
○	198
○	199
○	200
○	201
○	202
○	203
○	204
○	205
○	206
○	207
○	208
○	209
○	210
○	211
○	212
○	213
○	214
○	215
○	216
○	217
○	218
○	219
○	220
○	221
○	222
○	223
○	224
○	225

What is your body weight (kilograms)?

- ○ 25
- ○ 30
- ○ 31
- ○ 32
- ○ 33
- ○ 34
- ○ 35
- ○ 36
- ○ 37
- ○ 38
- ○ 39
- ○ 40
- ○ 41
- ○ 42
- ○ 43
- ○ 44
- ○ 45
- ○ 46
- ○ 47
- ○ 48
- ○ 49
- ○ 50
- ○ 51
- ○ 52
- ○ 53
- ○ 54
- ○ 55
- ○ 56

- 57
- 58
- 59
- 60
- 61
- 62
- 63
- 64
- 65
- 66
- 67
- 68
- 69
- 70
- 71
- 72
- 73
- 74
- 75
- 76
- 77
- 78
- 79
- 80
- 81
- 82
- 83
- 84
- 85

- ○ 86
- ○ 87
- ○ 88
- ○ 89
- ○ 90
- ○ 91
- ○ 92
- ○ 93
- ○ 94
- ○ 95
- ○ 96
- ○ 97
- ○ 98
- ○ 99
- ○ 100
- ○ 101
- ○ 102
- ○ 103
- ○ 104
- ○ 105
- ○ 106
- ○ 107
- ○ 108
- ○ 109
- ○ 110
- ○ 111
- ○ 112
- ○ 113
- ○ 114

○	115
○	116
○	117
○	118
○	119
○	120
○	121
○	122
○	123
○	124
○	125
○	126
○	127
○	128
○	129
○	130
○	131
○	132
○	133
○	134
○	135
○	136
○	137
○	138
○	139
○	140
○	141
○	142
○	143

- ○ 144
- ○ 145
- ○ 146
- ○ 147
- ○ 148
- ○ 149
- ○ 150
- ○ 151
- ○ 152
- ○ 153
- ○ 154
- ○ 155
- ○ 156
- ○ 157
- ○ 158
- ○ 159
- ○ 160
- ○ 161
- ○ 162
- ○ 163
- ○ 164
- ○ 165
- ○ 166
- ○ 167
- ○ 168
- ○ 169
- ○ 170
- ○ 171
- ○ 172

O	173
O	174
O	175
O	176
O	177
O	178
O	179
O	180
O	181
O	182
O	183
O	184
O	185
O	186
O	187
O	188
O	189
O	190
O	191
O	192
O	193
O	194
O	195
O	196
O	197
O	198
O	199
O	200

Biological Sex:

- ○ Male
- ○ Female

Other, please specify

....

What country do you live in?

- ○ Australia
- ○ Brazil
- ○ Canada
- ○ New Zealand
- ○ United Kingdom
- ○ United States of America
- ○ Other

Please specify which country you live in?

....

....

....

....

State/Territory:

- ○ Australian Capital Territory
- ○ New South Wales
- ○ Northern Territory
- ○ Queensland
- ○ South Australia
- ○ Tasmania
- ○ Victoria
- ○ Western Australia

State:

- ○ Acre
- ○ Alagoas
- ○ Amapá
- ○ Amazonas
- ○ Bahia
- ○ Ceará
- ○ Distrito Federal
- ○ Espírito Santo
- ○ Goiás
- ○ Maranhão
- ○ Mato Grosso
- ○ Mato Grosso do Sul
- ○ Minas Gerais
- ○ Pará
- ○ Paraíba
- ○ Paraná
- ○ Pernambuco
- ○ Piauí
- ○ Rio de Janeiro
- ○ Rio Grande do Norte
- ○ Rio Grande do Sul
- ○ Rondônia
- ○ Roraima
- ○ Santa Catarina
- ○ São Paulo
- ○ Sergipe
- ○ Tocantins

Province:

- ○ Alberta
- ○ British Columbia
- ○ Manitoba
- ○ New Brunswick
- ○ Newfoundland and Labrador
- ○ Nova Scotia
- ○ Ontario
- ○ Prince Edward Island
- ○ Quebec
- ○ Saskatchewan

Region:

- ○ Auckland
- ○ Canterbury
- ○ Wellington
- ○ Waikato
- ○ Bay of Plenty
- ○ Manawatu-Wanganui
- ○ Otago
- ○ Hawke's Bay
- ○ Northland
- ○ Taranaki
- ○ Southland
- ○ Nelson
- ○ Gisborne
- ○ Marlborough
- ○ Tasman
- ○ West Coast

County:

- Aberdeenshire
- Angus
- Antrim
- Argyll & Bute
- Armagh
- Ayrshire
- Banffshire
- Bath and North East Somerset
- Bedfordshire
- Berkshire
- Berwickshire
- Blaenau Gwent
- Borders
- Bridgend
- Bristol
- Buckinghamshire
- Caerphilly
- Caithness
- Cambridgeshire
- Cardiff
- Carmarthenshire
- Ceredigion
- Cheshire
- Clackmannanshire
- Conwy
- Cornwall
- County Durham
- Cumbria

- ○ Denbighshire
- ○ Derbyshire
- ○ Devon
- ○ Dorset
- ○ Down
- ○ Dumfries & Galloway
- ○ Dunbartonshire
- ○ East Ayrshire
- ○ East Dunbartonshire
- ○ East Lothian
- ○ East Renfrewshire
- ○ East Riding of Yorkshire
- ○ East Sussex
- ○ Essex
- ○ Fermanagh
- ○ Fife
- ○ Flintshire
- ○ Gloucestershire
- ○ Greater London
- ○ Greater Manchester
- ○ Gwynedd
- ○ Hampshire
- ○ Herefordshire
- ○ Hertfordshire
- ○ Highland
- ○ Inverclyde
- ○ Isle of Anglesey
- ○ Isle of Wight
- ○ Isles of Scilly

- Kent
- Kincardineshire
- Lanarkshire
- Lancashire
- Leicestershire
- Lincolnshire
- Londonderry
- Merseyside
- Merthyr Tydfil
- Midlothian
- Monmouthshire
- Moray
- Neath Port Talbot
- Newport
- Norfolk
- North Ayrshire
- North Lanarkshire
- North Somerset
- North Yorkshire
- Northamptonshire
- Northumberland
- Nottinghamshire
- Orkney
- Oxfordshire
- Pembrokeshire
- Perth & Kinross
- Powy
- Renfrewshire
- Rhondda Cynon Taff

- Rutland
- Shetland
- Shropshire
- Somerset
- South Ayrshire
- South Gloucestershire
- South Lanarkshire
- South Yorkshire
- Staffordshire
- Stirlingshire
- Suffolk
- Surrey
- Swansea
- Torfaen
- Tyne & Wear
- Tyrone
- Vale of Glamorgan
- Warwickshire
- West Dunbartonshire
- West Lothian
- West Midlands
- West Sussex
- West Yorkshire
- Western Isles
- Wiltshire
- Worcestershire
- Wrexham

State:

- ○ Alabama
- ○ Alaska
- ○ Arizona
- ○ Arkansas
- ○ California
- ○ Colorado
- ○ Connecticut
- ○ Delaware
- ○ Florida
- ○ Georgia
- ○ Hawaii
- ○ Idaho
- ○ Illinois
- ○ Indiana
- ○ Iowa
- ○ Kansas
- ○ Kentucky
- ○ Louisiana
- ○ Maine
- ○ Maryland
- ○ Massachusetts
- ○ Michigan
- ○ Minnesota
- ○ Mississippi
- ○ Missouri
- ○ Montana
- ○ Nebraska
- ○ Nevada

- ○ New Hampshire
- ○ New Jersey
- ○ New Mexico
- ○ New York
- ○ North Carolina
- ○ North Dakota
- ○ Ohio
- ○ Oklahoma
- ○ Oregon
- ○ Pennsylvania
- ○ Rhode Island
- ○ South Carolina
- ○ South Dakota
- ○ Tennessee
- ○ Texas
- ○ Utah
- ○ Vermont
- ○ Virginia
- ○ Washington
- ○ West Virginia
- ○ Wisconsin
- ○ Wyoming

Postcode/Zip-code:

....

....

....

....

(2/10) Your history of Parkinson's Disease

Do you currently have a diagnosis of Parkinson's Disease, or Parkinsonism, by a medical professional (e.g. a movement disorders specialist, neurologist, or geriatrician)?

- O Yes
- O No

What age where you when you were diagnosed with Parkinson's disease (years)?

- ○ 18
- ○ 19
- ○ 20
- ○ 21
- ○ 22
- ○ 23
- ○ 24
- ○ 25
- ○ 30
- ○ 31
- ○ 32
- ○ 33
- ○ 34
- ○ 35
- ○ 36
- ○ 37
- ○ 38
- ○ 39
- ○ 40
- ○ 41
- ○ 42
- ○ 43
- ○ 44
- ○ 45
- ○ 46
- ○ 47
- ○ 48
- ○ 49

- ○ 50
- ○ 51
- ○ 52
- ○ 53
- ○ 54
- ○ 55
- ○ 56
- ○ 57
- ○ 58
- ○ 59
- ○ 60
- ○ 61
- ○ 62
- ○ 63
- ○ 64
- ○ 65
- ○ 66
- ○ 67
- ○ 68
- ○ 69
- ○ 70
- ○ 71
- ○ 72
- ○ 73
- ○ 74
- ○ 75
- ○ 76
- ○ 77
- ○ 78

○	79
○	80
○	81
○	82
○	83
○	84
○	85
○	86
○	87
○	88
○	89
○	90
○	91
○	92
○	93
○	94
○	95
○	96
○	97
○	98
○	99
○	100
○	101
○	102
○	103
○	104
○	105
○	106
○	107

- ○ 108
- ○ 109
- ○ 110
- ○ 111
- ○ 112
- ○ 113
- ○ 114
- ○ 115
- ○ 116
- ○ 117
- ○ 118
- ○ 119
- ○ 120+

What age where you when you first noticed symptoms of Parkinson's disease (years)?

- ○ 18
- ○ 19
- ○ 20
- ○ 21
- ○ 22
- ○ 23
- ○ 24
- ○ 25
- ○ 30
- ○ 31
- ○ 32
- ○ 33
- ○ 34
- ○ 35
- ○ 36
- ○ 37
- ○ 38
- ○ 39
- ○ 40
- ○ 41
- ○ 42
- ○ 43
- ○ 44
- ○ 45
- ○ 46
- ○ 47
- ○ 48
- ○ 49

O	50
O	51
O	52
O	53
O	54
O	55
O	56
O	57
O	58
O	59
O	60
O	61
O	62
O	63
O	64
O	65
O	66
O	67
O	68
O	69
O	70
O	71
O	72
O	73
O	74
O	75
O	76
O	77
O	78

- ○ 79
- ○ 80
- ○ 81
- ○ 82
- ○ 83
- ○ 84
- ○ 85
- ○ 86
- ○ 87
- ○ 88
- ○ 89
- ○ 90
- ○ 91
- ○ 92
- ○ 93
- ○ 94
- ○ 95
- ○ 96
- ○ 97
- ○ 98
- ○ 99
- ○ 100
- ○ 101
- ○ 102
- ○ 103
- ○ 104
- ○ 105
- ○ 106
- ○ 107

- ○ 108
- ○ 109
- ○ 110
- ○ 111
- ○ 112
- ○ 113
- ○ 114
- ○ 115
- ○ 116
- ○ 117
- ○ 118
- ○ 119
- ○ 120+

Are you currently taking prescription medication for treating the symptoms of Parkinson’s disease?

- ○ Yes
- ○ No

If yes, please specify which medication/s you are taking (tick all that apply)

- ☐ Lodosyn (Carbidopa)
- ☐ Sinemet (Carbidopa-Levodopa Oral)
- ☐ Sinemet CR (Carbidopa-Levodopa Oral, Extended Release)
- ☐ Stalevo (Carbidopa, Levodopa, And Entacapone)
- ☐ Azilect (Rasagiline)
- ☐ Madopar (Levodopa-Benserazide)
- ☐ Neupro (Rotigotine)
- ☐ Sifrol, Mirapex or Mirapexin (Pramipexole)
- ☐ Sifrol ER or Mirapex ER (Pramipexole)
- ☐ Duopa or Duodopa (Carbidopa-Levodopa Intestinal Gel)

Other, please specify

....

Is your Parkinson's disease currently treated by Deep Brain Stimulation (DBS)?

- ○ Yes
- ○ No

Have you been diagnosed with any of the following medical conditions (Tick all that apply):

- ☐ Heart disease
- ☐ High blood pressure
- ☐ Diabetes
- ☐ Lung disease (not cancer)
- ☐ Kidney disease (not cancer)
- ☐ Liver disease (not cancer)
- ☐ Cancer
- ☐ Depression
- ☐ Anxiety
- ☐ Arthritis

Other, please specify:

....

Are you taking prescription medication to treat your condition(s)?

- ○ Yes
- ○ No

Please write the name of the medication(s) you are taking.

....

....

....

....

Have you become more sensitive to the heat with Parkinson's Disease?

- ○ Yes
- ○ No

(3/10) General Parkinson's experiences This section asks you about your general health over the last four weeks. Please read each statement carefully and rate your experience as either never, occasionally, sometimes, often, or always.

Over the past four weeks have you, because of your Parkinson's Disease…

Had difficulty getting around in public places?

	Never	Occasionally	Sometimes	Often	Always (or cannot do at all)
	○	○	○	○	○

Had difficulty dressing yourself?

	Never	Occasionally	Sometimes	Often	Always (or cannot do at all)
	○	○	○	○	○

Felt depressed?

	Never	Occasionally	Sometimes	Often	Always (or cannot do at all)
	○	○	○	○	○

Had problems with close relationships?

	Never	Occasionally	Sometimes	Often	Always (or cannot do at all)
	○	○	○	○	○

Had problems with concentration?

	Never	Occasionally	Sometimes	Often	Always (or cannot do at all)
	○	○	○	○	○

Felt unable to communicate properly?

	Never	Occasionally	Sometimes	Often	Always (or cannot do at all)
	○	○	○	○	○

Had painful muscle cramps and pains?

	Never	Occasionally	Sometimes	Often	Always (or cannot do at all)
	○	○	○	○	○

Over the past four weeks have you felt embarrassed by having Parkinson’s Disease?

	Never	Occasionally	Sometimes	Often	Always (or cannot do at all)
	○	○	○	○	○

(4/10) Heat Specific Parkinson's Experiences

As a person with Parkinson's Disease, what happens to you when you get too hot? (Answer yes/no to each statement):

Nothing, I cope just fine

- ○ Yes (Agree)
- ○ No (Disagree)

I lack energy and require more rest.

- ○ Yes
- ○ No

Apart from fatigue, my other symptoms of Parkinson's Disease become worse.

- ○ Yes
- ○ No

I am unable to participate in my usual social activities (time with family or friends).

- ○ Yes
- ○ No

I am unable to do my usual household duties (e.g. cleaning, cooking, etc.).

- ○ Yes
- ○ No

I am unable to work effectively.

- ○ Yes
- ○ No

I am unable to look after myself in the usual manner.

- ○ Yes
- ○ No

I need more medication to cope.

- ○ Yes
- ○ No

I have felt sufficiently unwell to require a doctor or other health professional.

- ○ Yes
- ○ No

I have been hospitalised because of heat.

- ○ Yes
- ○ No

(5/10) Use of Air Conditioning

Do you use an air conditioner at home to keep cool on hot days or nights?

- ○ Yes
- ○ No

I do not use an air conditioner at home because... (Please check all applicable reasons)

- ☐ I do not have a problem with hot weather.
- ☐ I do have a problem with hot weather but cannot afford to buy an air conditioner.
- ☐ I have an air conditioner and need to use it, but cannot afford the electricity costs.
- ☐ I have an air conditioner and need to use it, but it is broken

How hot is it outside when you usually turn on your air conditioner?

- ☐ 20-24 ° C (68 - 75.2 ° F)
- ☐ 25- 29 ° C (77 - 84.2 ° F)
- ☐ 30-34 ° C (86 - 93.2 ° F)
- ☐ 35 -39 ° C (95 - 102.2 ° F)

What temperature do you set your air conditioner?

- ○ Celsius
- ○ Fahrenheit

Please specify temperature:

....

Do you receive a subsidy or rebate for cooling your home?

- ○ Yes
- ○ No

(6/10) Your Experience of the Heat

The next sections of the questionnaire (Parts 6, 7, 8, and 9) will ask you about your experiences of heat. The researchers are trying to be thorough, so some of the statements may not apply to you now or ever. Please read each statement carefully and rate your experience as either never, rarely, sometimes, often, or always. Comfort and sensation of heat.

I usually prefer a cooler air temperature compared to other people my age.

	Never	Rarely	Sometimes	Often	Always
	○	○	○	○	○

I usually prefer a warmer air temperature compared to other people my age.

	Never	Rarely	Sometimes	Often	Always
	○	○	○	○	○

My body temperature fluctuates (goes up and down) more than I would like during the day-time .

	Never	Rarely	Sometimes	Often	Always
	○	○	○	○	○

My body temperature fluctuates (goes up and down) more than I would like during the night -time .

	Never	Rarely	Sometimes	Often	Always
	○	○	○	○	○

What other people my age would describe as a comfortable summer day, I feel is too hot .

	Never	Rarely	Sometimes	Often	Always
	○	○	○	○	○

What other people my age would describe as a comfortable summer day, I feel is too cold .

	Never	Rarely	Sometimes	Often	Always
	○	○	○	○	○

(7/10) The way in which the heat affects your daily activities

I try to avoid getting hot.

	Never	Rarely	Sometimes	Often	Always
	○	○	○	○	○

I avoid my usual daily activities on a hot day.

	Never	Rarely	Sometimes	Often	Always
	○	○	○	○	○

I have difficulty looking after my home and doing housework due to the heat.

	Never	Rarely	Sometimes	Often	Always
	○	○	○	○	○

I have difficulty sleeping due to the heat.

	Never	Rarely	Sometimes	Often	Always
	○	○	○	○	○

I have difficulty attending social events (e.g. time with family and friends) due to the heat.

	Never	Rarely	Sometimes	Often	Always
	○	○	○	○	○

I have difficulty doing physical activity and/or exercise because it makes me feel too hot.

	Never	Rarely	Sometimes	Often	Always
	○	○	○	○	○

I have been confined to my house more than I would like due to the heat.

	Never	Rarely	Sometimes	Often	Always
	○	○	○	○	○

I worry about how I will cope with the heat.

	Never	Rarely	Sometimes	Often	Always
	○	○	○	○	○

Feeling hot makes it difficult for me to concentrate.

	Never	Rarely	Sometimes	Often	Always
	○	○	○	○	○

(8/10) The effects of heat on your body

I have difficulty tolerating (coping with) the heat.

	Never	Rarely	Sometimes	Often	Always
	○	○	○	○	○

The heat makes me feel tired.

	Never	Rarely	Sometimes	Often	Always
	○	○	○	○	○

I have less energy on hot days.

	Never	Rarely	Sometimes	Often	Always
	○	○	○	○	○

The heat makes my mood worse.

	Never	Rarely	Sometimes	Often	Always
	○	○	○	○	○

A comfortable summer day for other people my age becomes a debilitating challenge for me.

	Never	Rarely	Sometimes	Often	Always
	○	○	○	○	○

It takes me longer to cool down after physical activity and/or exercise compared to other people my age.

	Never	Rarely	Sometimes	Often	Always
	○	○	○	○	○

I sweat too much in the heat.

	Never	Rarely	Sometimes	Often	Always
	○	○	○	○	○

Please specify which body areas you experience too much sweating.

- ☐ Whole body
- ☐ Head
- ☐ Face
- ☐ Neck
- ☐ Upper Back
- ☐ Lower Back
- ☐ Upper Arms
- ☐ Lower Arms
- ☐ Hands
- ☐ Chest
- ☐ Abdomen
- ☐ Pelvis
- ☐ Thighs
- ☐ Lower Legs
- ☐ Feet

Excessive sweating affects my relationships with others (e.g. having to sleep in different beds; body odour)

	Never	Rarely	Sometimes	Often	Always
	○	○	○	○	○

I usually start sweating in cooler air temperatures compared to other people my age.

	Never	Rarely	Sometimes	Often	Always
	○	○	○	○	○

I usually start sweating earlier during exercise and/or physical activity compared to other people my age.

	Never	Rarely	Sometimes	Often	Always
	○	○	○	○	○

I talk to my medical professional about how the heat affects me.

	Never	Rarely	Sometimes	Often	Always
	○	○	○	○	○

My movement symptoms of Parkinson's Disease get worse in the heat.

	Never	Rarely	Sometimes	Often	Always
	○	○	○	○	○

Which symptoms get worse in the heat   Check all boxes that apply.

- ☐ Tremor (shaking)
- ☐ Freezing while walking
- ☐ Stiffness
- ☐ Difficulty walking
- ☐ Walking Problems
- ☐ Balance Problems
- ☐ Problems Chewing and Swallowing
- ☐ Difficulty dressing

If any others, please specify:

....

My non-movement symptoms of Parkinson’s disease get worse in the heat (e.g.) fatigue, depression

	Never	Rarely	Sometimes	Often	Always
	○	○	○	○	○

Which symptoms get worse in the heat (check all that apply):

- ☐ Fatigue
- ☐ Constipation
- ☐ Frequent urination
- ☐ Urgent urination
- ☐ Anxiety
- ☐ Depression
- ☐ Difficulty concentrating
- ☐ Memory problems (forgetfulness)
- ☐ Difficulty Sleeping
- ☐ Loss of taste or smell
- ☐ Difficulty swallowing
- ☐ Sweating
- ☐ Dribbling of saliva
- ☐ Light-headedness when standing

Other, please specify:

....

My Parkinson's medication is less effective in the heat.

	Never	Rarely	Sometimes	Often	Always
	○	○	○	○	○

My OFF periods (times when medication is less effective) are made worse by the heat.

	Never	Rarely	Sometimes	Often	Always
	○	○	○	○	○

(9/10) Symptoms in the Heat

I feel lightheaded in the heat.

	Never	Rarely	Sometimes	Often	Always
	○	○	○	○	○

I feel weak in the heat.

	Never	Rarely	Sometimes	Often	Always
	○	○	○	○	○

I feel dizzy in the heat.

	Never	Rarely	Sometimes	Often	Always
	○	○	○	○	○

I feel unsteady on my feet in the heat.

	Never	Rarely	Sometimes	Often	Always
	○	○	○	○	○

I feel nauseous in the heat.

	Never	Rarely	Sometimes	Often	Always
	○	○	○	○	○

I feel disorientated in the heat.

	Never	Rarely	Sometimes	Often	Always
	○	○	○	○	○

(10/10) Any other comments.

Please describe in your own words how the heat affects you (optional):

....

....

....

....

Did you have help filling out this questionnaire?

○ No, I filled it in myself

○ Yes, someone else helped me

Thank you very much for completing the questionnaire. Your answers are helping researchers and clinicians to better understand the ways in which the heat affects people living with Parkinson's disease.
